# What Do We Know About Rural Mobile Health Clinics? A Scoping Review

**DOI:** 10.3390/ijerph23050558

**Published:** 2026-04-25

**Authors:** Katherine Simmonds, Madison Evans, Nancy Nguyen, Niharika Putta, Alexis Thom

**Affiliations:** 1 Institute for Health Equity and Social Justice Research, Bouvé College of Health Sciences, Boston Campus, Northeastern University, Boston, MA 02115, USA; evans.madi@northeastern.edu (M.E.); nguyen.nanc@northeastern.edu (N.N.); thom.a@northeastern.edu (A.T.); 2 School of Nursing, Northeastern University, Boston, MA 02115, USA; 3 The Roux Institute, Northeastern University, Portland, ME 01401, USA; 4 Department of Undergraduate Medical Education, University of Massachusetts Chan Medical School, 55 N Lake Ave, Worcester, MA 01655, USA; niharika.putta@umassmed.edu

**Keywords:** mobile health clinics, rural health, healthcare access, health disparities, primary care delivery, patient outcomes, cost-effectiveness, healthcare sustainability, underserved populations, community health

## Abstract

**Highlights:**

**Public health relevance—How does this work relate to a public health issue?**

- Growing rural–urban health disparities in life expectancy, chronic disease, and maternal and infant mortality are driven in part by structural barriers to healthcare access, including geographic isolation and transportation gaps.
- Mobile health clinics (MHCs) are a promising strategy for addressing these barriers, yet evidence about whether they increase access, improve patient outcomes, and are financially sustainable in rural settings has not been systematically investigated.

**Public health significance—Why is this work of significance to public health?**

- This review is the first to synthesize evidence on patient access, health outcomes, and financial sustainability among rural MHCs.
- While the literature suggests rural MHCs have potential to improve healthcare access, significant gaps exist in the available research and program evaluations.

**Public health implications—What are the key implications or messages for practitioners, policy makers and/or researchers in public health?**

- Rural MHCs are most viable when designed as comprehensive, multi-service programs supported by diverse funding, strong community engagement, and technological integration; per-visit costs rise with lower population density and longer travel distances and must be factored into program planning.
- Policy makers should sustain investment in rural MHC programs as existing evidence suggests they increase access and are acceptable; simultaneously, resources should be directed to support more rigorous evaluation that measures improvements in access, clinical outcomes and cost-effectiveness.

**Abstract:**

Rural communities face significant healthcare access barriers that contribute to persistent health disparities. Mobile health clinics (MHCs) have emerged as a promising strategy for expanding healthcare access, yet their effectiveness in rural settings remains understudied. The aim of this review was to examine the literature to determine what is known about access, health outcomes, and the cost-effectiveness of rural MHCs, specifically with regard to their impact on patient access and outcomes, return on investment (ROI)/financial, and program sustainability. We conducted a comprehensive search of peer-reviewed and grey literature sources. Systematic screening yielded 34 documents for full analysis. Thematic analysis was conducted across three domains: patient access, patient outcomes, and ROI/sustainability. All 34 documents provided data on patient access, with common themes including expanded service utilization, multi-service integration, overcoming geographic and transportation barriers, and improved healthcare affordability. Thirty-two documents addressed patient outcomes, reporting improvements in preventive care delivery, chronic disease management, and high patient satisfaction. Twenty-eight documents included ROI/sustainability information, with evidence suggesting cost-effectiveness particularly through emergency department visit avoidance and multi-service integration. Across the literature reviewed, the quality of evidence varied considerably, yet we concluded mobile health clinics demonstrate promise for expanding healthcare access and improving outcomes in rural populations. Key success factors include multi-service integration, diverse funding partnerships, technological integration, and strong community engagement. More rigorous research with longitudinal clinical outcome measures and robust economic analyses is needed.

## 1. Introduction

Rural communities in the United States face longstanding challenges with accessing quality healthcare. This has contributed to marked health disparities between rural and urban populations, including in life expectancy, maternal and infant mortality, and numerous other health conditions [1,2,3]. Higher rates of death in rural as compared with urban populations during the COVID-19 pandemic further exposed the urgent need for innovative and tailored solutions to address the unique health challenges people in these communities face [4]. Government agencies, such as the Department of Veterans Affairs and the Advanced Research Projects Agency for Health (ARPA-H) have responded to this identified need by prioritizing rural healthcare through targeted funding and initiatives that emphasize the development of scalable and effective interventions.

Mobile health clinics (MHCs) have been garnering growing attention in the United States and globally as a promising healthcare delivery strategy for bridging gaps in access, especially among rural and other hard-to-reach populations. Although MHCs have been shown to operate successfully in a variety of settings, particularly many urban areas, their impact in rural communities is less well understood. Key questions about whether MHCs expand access to care, improve patient outcomes, and offer a cost-effective approach to healthcare delivery in rural areas are important for determining whether the investment of time, money and other resources into such programs is warranted. The aim of this literature review is to examine current evidence on whether MHCs bridge gaps in primary care access and improve health outcomes in rural populations, as well as on the return on investment (ROI) and financial and program sustainability.

## 2. Background

Populations adversely affected by rising healthcare costs and high rates of chronic disease are typically poorly served by traditional healthcare delivery approaches in the United States [5]. Rural communities are among those underserved, and a growing disparity in urban–rural mortality since the 1980s provides stark evidence of this trend. Since 2012, urban mortality rates have plateaued, but in rural populations, life expectancy continues to decline. Chronic conditions such as cardiovascular and lower respiratory disease contribute to this disparity [6,7]; however, differences in the prevalence of chronic diseases do not completely explain this urban–rural mortality gap. Inequities in access to quality healthcare due to structural barriers including lack of health insurance/ability to pay for healthcare, language, physical and mental disabilities, inflexible work schedules, geography, weather, transportation, and provider shortages, are also known contributors to these poorer health outcomes [8].

Primary care, defined as “the provision of integrated, accessible health care services by clinicians who are accountable for addressing a large majority of personal health care needs, developing a sustained partnership with patients, and practicing in the context of family and community” [9], has been shown to improve health outcomes by offering a consistent source of care, early detection and treatment of disease, chronic disease management, and preventive care, such as vaccinations, and blood pressure and cancer screenings. In rural areas, severe primary care provider shortages impede access to these beneficial health services [10].

While healthcare has typically been delivered via centrally located brick-and-mortar facilities in recent years, these settings can be suboptimal for rural populations because of the previously mentioned factors including long distances to facilities and lack of transportation (private or public). Mobile health clinics or units (MHCs or MHUs) are an alternative approach to care delivery in communities experiencing geographic, social, or economic barriers. Khanna and Narula define an MHU as “a custom vehicle loaded with equipment, medical supplies, and skilled professionals, all set to bring the healthcare, which is otherwise inaccessible, closer to where people live and work” [11]. Across various definitions, a common theme is that these alternative care delivery models play a unique role in reaching vulnerable populations, including those with complex conditions who may not be able to effectively navigate the traditional healthcare system. As these terms appear to be interchangeable in the literature, hereafter we have elected to use the abbreviation MHC.

As of 2025, approximately 3000 MHCs were in operation in the U.S., providing some 10 million annual visits [12]. MHCs have demonstrated impressive returns on investment (ROI), beneficial patient outcomes, and the ability to reach vulnerable populations [13]. The Harvard Medical School’s Family Van is an exemplar of the potential financial impact of an MHC, with an estimated $3,125,668 in costs for Emergency Department visits averted as a result of services delivered in 2009 [13,14,15]. Broadly, research has indicated MHCs are effective in increasing access to healthcare and improving patient outcomes, and can be financially sustainable; however, this literature is heavily weighted toward their use in urban populations and in the delivery of specialty services (i.e., mammography, vision screening, dental care, etc.) [1]. A review of the literature that explores the effectiveness of MHCs as a strategy for delivering preventive and primary care services specifically in rural settings has not previously been published, signaling a gap that should be addressed to inform decision making and resource allocation. This review aims to explore the following research question: What is known about access, health outcomes, and the cost-effectiveness of rural MHCs? Specifically, we sought to identify existing evidence on whether mobile health clinics: (a) increase access to care; (b) improve patient outcomes; and (c) can support the return on investment (ROI) and be financially and programmatically sustainable in rural populations.

## 3. Materials and Methods

### 3.1. Database Selection

We conducted a comprehensive literature review between February and April 2025 within four primary databases: PubMed, Social Science Premium Collection, Business Source Complete, and ProQuest Healthcare Administration. These databases were chosen to ensure a broad, interdisciplinary set of perspectives on the effectiveness of MHCs in rural healthcare delivery. Together, literature from these sources enabled a comprehensive review of the existing published peer-reviewed literature on mobile health programs in rural settings, yielding both empirical data and operational insights across clinical and economic contexts.

#### Search Strategy

To identify studies aligned with our research question, we developed a structured search strategy using both MeSH terms and keyword variations tailored to each database. Search terms were designed to capture the literature focused on mobile health delivery in rural populations, with a specific emphasis on access to care, patient outcomes, and financial and programmatic sustainability.

In PubMed, we employed the following MeSH-based terms to generate an initial body of articles: (“Rural Health” [MeSH Terms] OR “Rural Population” [MeSH Terms]) AND (“Mobile Health Units” [MeSH Terms] OR “mobile health unit”* OR “mobile health clinic”*) AND “Health Services Accessibility” [MeSH Terms] AND “Delivery of Health Care” [MeSH Terms]. We also used variations such as: (“Rural Health” [MeSH Terms] OR “Rural Population” [MeSH Terms]) AND (“Mobile Health Units” [MeSH Terms] OR “mobile health unit”* OR “mobile health clinic”*). To broaden our search and capture studies with diverse methodological frameworks, we also searched using keyword combinations such as: (“Rural Health” [MeSH Terms] OR “Rural Population” [MeSH Terms]) AND (“Mobile Health Units” [MeSH Terms] OR “mobile health unit” OR “mobile health clinic”). To eliminate MHC programs that deliver specialty services exclusively, these terms were combined with NOT (mammogram OR mammography OR breast OR asthma OR behavioral health OR psychiatric OR psychology OR mental health OR mental illness OR dental OR dentist OR oral OR (maternal AND infant) OR (maternal AND child) OR pediatrics OR child OR infant OR natal OR neonatal OR sexual OR reproductive OR vision OR eye).

Searches in the Social Science Premium Collection, Business Source Complete, and ProQuest Healthcare Administration used equivalent keyword structures adapted to each platform’s indexing system. We included terms such as “mobile medical unit,” “rural healthcare access,” “health equity,” “economic sustainability,” and “community-based health services.” A librarian from the Northeastern University Library system was consulted to guide the search process.

All searches were limited to articles published in English since 2009. Both U.S.-based and international mobile health programs were included. Although we recognize that healthcare systems are financed and organized in fundamentally different ways across countries, our decision to include literature from outside the US stems from our belief that other countries may offer important insights about this mode of healthcare delivery and about addressing the healthcare needs of rural populations, which could be transferable. Additionally, our inclusion criteria required the mobile health clinic program to serve a rural population and deliver general healthcare services, such as primary care, chronic disease management, health screenings, or vaccinations. We excluded programs that only offered specialty services (e.g., only mammography, HIV care, dental services, or mental health/substance use disorder services). However, programs that integrated such services into a broader primary care model were retained for full-text review. This search strategy yielded a preliminary set of articles that were uploaded to Covidence for further screening, described further below.

### 3.2. Grey Literature Search

In addition to the peer-reviewed literature search, we conducted a thorough search of grey literature to identify other relevant sources on rural mobile health programs. We began with a list of self-reported mobile health clinics from the Mobile Health Map database (n = 552) (database publicly accessible at https://www.mobilehealthmap.org/clinics/, accessed on 25 March 2025), which included information on services provided, type of community and populations served, website links, and clinic addresses. Based on our predetermined inclusion and exclusion criteria, we first selected clinics that self-identified as serving exclusively rural, frontier, or rural/frontier communities, and excluded those operating in suburban or urban settings. MHCs that provided only specialized services (e.g., mammography, dental-only services) were also removed from the list. The remaining programs were then screened for eligibility through a two-step process. First, we carried out a preliminary verification of clinic type and activity using an AI-based tool (ChatGPT version 4.1). Second, a reviewer manually confirmed each clinic’s status, with uncertain cases reviewed by a second reviewer (KS) or discussed with the research team during a meeting. A total of 106 programs met our inclusion criteria, of which 93 were based in the United States and 13 were international. Given the large initial number of mobile health programs, this AI-based search made it more feasible for us to identify potential sources of relevant information, which we subsequently confirmed and expanded through manual searches.

After finalizing our list of eligible MHCs, we combined both AI-assisted and manual Google searches to identify relevant grey literature—annual reports, program evaluations, news articles, policy briefs, webpages, and other non-peer-reviewed materials—that addressed at least one of our three primary research questions. Each of the identified artifacts was then documented in an evidence table, noting publication type, date, location of clinic, and whether it addressed the research question(s) with quantitative or qualitative data. All grey literature sources were uploaded to Zotero for reference management, and relevant documents were imported into Covidence to facilitate team-based screening alongside the peer-reviewed literature review.

### 3.3. Screening and Review Process

A total of 321 peer-reviewed and grey literature articles were initially uploaded into Covidence for systematic screening. After removing 44 duplicates, 277 articles/artifacts remained. Titles and abstracts were independently reviewed by two team members to assess relevance against our predefined inclusion and exclusion criteria. Articles that were irrelevant or did not meet our criteria, such as those not focusing on MHCs, not serving rural populations, or outside the date range, were excluded, resulting in a total of 127 articles for full-text review.

During full-text review, studies were further screened for relevance by two team members, including to confirm relevance to at least one of our research foci. To remain in the final group of articles for full-text review, we also verified that the mobile health program was delivered entirely or in some part out of some type of vehicle (in contrast to programs operating out of a non-permanent, non-clinical space, such as a community center, church, or school, or those that were solely digitally based). Differences in team members’ assessment of whether to include an article/artifact in the final review were brought to the entire study team for further discussion and resolution. Articles were excluded if they did not mention MHCs, focused exclusively on specialty services, or had no data on patient access, outcomes, or ROI/financial sustainability. Following this step, 34 studies remained for detailed data extraction. Covidence was used to systematically categorize information pertinent to the research questions under the groupings of patient outcomes, patient access, and economic sustainability/ROI. We also created an additional category of innovative program structures and operational insights, the results of which are beyond the scope of this paper.

A PRISMA flow diagram (Appendix A) documents the selection process, including total records retrieved, duplicates removed, records screened by title and abstract, full-text articles assessed, and studies included in the final analysis. This systematic approach ensured that only studies meeting the defined criteria for rural mobile health clinics and with data relevant to our research questions were included. Approximately half of the articles/artifacts (hereafter referred to as “documents”) retained for full review were from peer-reviewed journals, while the remainder were items such as annual reports, news stories, and webpages. (See Table S1: Preferred Reporting Items for Systematic reviews and Meta-Analyses extension for Scoping Reviews (PRISMA-ScR) Checklist for additional information about this search). 

## 4. Results

Our review of the literature retained following a systematic search of the formal and grey literature outlined in the previous section revealed that all 34 documents included data on patient access, 32 had information on patient outcomes, and 28 presented some type of data on ROI/program sustainability. Fifteen of the documents were based on MHC programs in the United States, while the other 19 operated in countries outside the US (see Table 1).(See Table S2: Characteristics of Sources of Evidence for additional information about this literature). 

We carried out a thematic analysis of this literature, which we first organized broadly according to our three research question categories: patient access, patient outcomes, and the sustainability/ROI of the MHC program. Emergent themes within each of these categories were then identified and are described below.

### 4.1. Patient Access

Expanded patient access to healthcare services is a frequently cited rationale for mobile health programs. Among the 34 documents reviewed, all included some type of data about how the program increased patient access to care. Within this category, four emergent themes were identified, including: (i) patient visits and service utilization; (ii) multi-service integration; (iii) overcoming geographic, transportation, and infrastructure barriers; and (iv) healthcare affordability.

#### 4.1.1. Theme 1: Patient Visits and Service Utilization

Much of the quantitative data on patient access in the literature reviewed consisted of the number of patient visits to the MHC program during a given period (typically a year or month). For example, Augusta Health Neighborhood Clinics reported having a “significant impact” by providing over 1700 primary care visits for 825 patients in 17 communities during its first year of full operation [16]. Similarly, the Kuruman Community Trust Mobile Medical Clinic, which operates as a non-profit in collaboration with the North Cape Department of Public Health in South Africa, reports their mobile clinic model is a “successful way to provide healthcare services to remote communities” and notes 70,000 patient visits over five years of operation as a key achievement that supports this claim [17]. The ubiquitousness of data on patient visits across this literature suggests it is a priority metric among rural MHC programs around the world. We noted wide variation in the volume of patient visits across the different MHC programs represented, with some reporting several hundred and others tens of thousands of patient visits annually. This range appeared to be related to a variety of operational factors, including the density of the population served, proximity of the population(s) to other healthcare providers, and availability of funding and vehicles for program use, as well as other specific characteristics about the MHC program, such as length of time in operation, reputation and community trust. We noted most of the documents suggested or conflated patient visits with increased access to healthcare, but did not provide specific data to support this assertion.

In addition to enumerating patient visits, service utilization was also reported as an indicator of increased access in some of the documents reviewed. While this metric provides a more nuanced accounting of the actual types of services delivered as compared to patient visit volume, it also equates services delivered with increased access without substantive data to support this assertion. One notable exception to this was a peer-reviewed research article that included detailed data on service utilization [18]. The MHC program, Right to Care, provided reproductive and primary care via a nurse clinician in two rural districts in South Africa. Total patient encounters, as well as pap smear screenings, HIV counseling and testing, contraceptive services, condom distribution, breast examinations, and sexually transmitted infection/candidiasis treatment were reported. This detailed accounting enabled a cost analysis to be calculated for each type of service in both delivery sites. Such service-specific volume reporting allows granular analysis of service utilization and comparison to identified population health needs, building an evidence base for claims about increased access. This type of quantitative research that links service utilization with cost-effectiveness and access stood out among the documents reviewed.

#### 4.1.2. Theme 2: Multi-Service Integration

Another theme noted across the literature reviewed was the practice of bundling multiple health services within rural MHC programs. Rather than single-purpose offerings, many documents described program models that offered a variety of services including chronic disease management, infectious disease screening, reproductive health, behavioral health, and preventive care. Unlike urban settings where high-density populations can support MHC programs with a narrow or single focus, such as mammograms or HIV screening and treatment, many rural populations have limited access to any type of healthcare, and therefore patients often present seeking care for more than one issue.

The previously mentioned Right to Care program offers one example of a bundled care approach to rural healthcare delivery through an MHC program [18]. Initially established to increase cervical cancer screening among rural women in South Africa, in practice both of its MHCs provided a range of health services, including HIV/AIDS screening and treatment, STIs and tuberculosis screening, breast exams, blood pressure monitoring, and health education, as well as care for men. Among the MHCs represented in this review, such service integration strategies appeared to be deliberate rather than opportunistic, recognizing the needs and benefits for rural populations of comprehensive services that can address multiple health issues simultaneously. This finding suggests that multi-service integration represents both a clinical best practice as well as a strategy for economic sustainability (discussed further below). Our finding that multi-service integration was common among programs represented in our review indicates it has been broadly adopted as a standard rather than innovative approach among rural MHC programs.

#### 4.1.3. Theme 3: Overcoming Geographic, Transportation and Infrastructure Barriers

In many of the documents reviewed, MHC programs were described as a strategy for addressing geographic barriers to healthcare. As one example, the mobile units deployed by the Order of Malta in Lebanon serve both Syrian refugees in the rural area of Beqaa who lack transportation, as well as the local Lebanese population [19]. Some documents also mentioned MHC programs bridged transportation barriers and broadband/Wi-Fi infrastructure gaps to enable telehealth visits. Geographic barriers were primarily characterized as “long distances” or “remote” areas without healthcare facilities, but some documents were more specific with actual travel times or measures of distance to the nearest existing brick-and-mortar health facility reported [20]. One reported the MHC went directly to the mines to deliver services to the target population (miners) to overcome distance, transportation, and occupational barriers, such as long work hours [21]. Similarly, some MHC programs that target agricultural workers bridged both distance and occupational constraints by bringing services to locations where people work. Other programs, including ones serving unhoused populations in Michigan, displaced families in Lebanon, and rural communities across Africa, asserted that bringing care directly into neighborhoods, shelters, or schools reduced barriers [22,23,24].

In addition to distance, many of the documents mentioned increasing access to care by addressing lack of transportation. One MHC program in Sub-Saharan Africa noted poor road infrastructure as a transportation-related barrier to access [24]. Additionally, several documents mentioned expanded access to telehealth by incorporating this capacity into the MHC, giving patients access to both routine and specialty care that otherwise would not be available [21,25]. Additionally, one document noted this infrastructure feature expanded program capacity by enabling tele-mentorship for providers delivering care out of the MHC [21].

#### 4.1.4. Theme 4: Healthcare Affordability

In the literature reviewed, quite a few documents mentioned that services delivered out of the MHC were provided at no or low cost to patients. Several mentioned that the patients served by the program were un- or underinsured [26,27,28,29,30,31]. Garnelo and colleagues [32] noted that while fluvial mobile units increased access to primary care for people in the Amazon, costs associated with getting to the unit and of pursuing recommended follow up specialty services in urban centers were barriers for many. 

One document described the use of care navigators to assist patients with connecting to other social services and resources [27], and another mentioned distribution of affordable food along with free health screenings as part of their model [33]. Overall, the literature indicated that most rural MHC programs serve patients for whom paying for health services poses a significant barrier. Together with distance and transportation barriers, reduction or elimination of payment for healthcare services was identified as a common strategy for increasing access to care among rural MHCs.

### 4.2. Patient Outcomes

Thirty-two of the 34 documents reviewed included some type of data on patient outcomes; however, the types and rigor of the reported outcome measures varied widely across this literature. Nevertheless, several overarching themes emerged, including: (1) improvements in clinical and preventive health outcomes; (2) high patient satisfaction and perceived quality of care; and (3) broader individual and community-level benefits. Each of these themes is discussed below.

#### 4.2.1. Theme 1: Clinical and Preventive Health Outcome Improvements

In the literature reviewed, preventive care, such as vaccinations administered and chronic disease management visits, and early detection screenings, were commonly reported indicators of improved clinical and health outcomes among rural MHCs. As one example, the Clemson Rural Health Impact Report, which included data from their MHC program, reported conducting over 12,283 health screenings, reaching 1490 patients across rural counties between 1 July 2021 to 30 June 2022, with improvements in chronic disease indicators including diabetes and hypertension [34]. Of note, however, though the MHC program served 127 locations, the impact report did not distinguish how many of these health screenings were delivered via the MHC versus the program’s brick-and-mortar clinics. As another example, the Traverse City Mobile Medical Unit reported providing nearly 1000 visits with 400 individuals supplied with urgent care, chronic disease management, and behavioral health support, and linked these to stabilizing conditions such as diabetes, hypertension, and asthma [22]. This type of non-specific data reporting connecting patient visits with improved clinical outcomes was common across the grey literature reviewed.

Improved maternal and reproductive health outcomes were also reported by several programs. During a 3-month initiative (August to October 2022), mobile antenatal clinics in Niger served 2923 pregnant women and provided 18,708 vaccines to children under 1 year old [35]. Similarly, a mobile clinic program in Mysore, India, successfully integrated antenatal care and screening for HIV, syphilis, hepatitis B, bacterial vaginosis, anemia, hypertension, urine albumin, and blood sugar to 3545 pregnant women [36]. The program identified and treated 22 HIV cases, 19 hepatitis B cases, 2 syphilis cases, and 250 cases of bacterial vaginosis, enabling early intervention to reduce maternal complications and risks of mother-to-child transmission. Additionally, 1755 women (49.5%) were diagnosed with moderate to severe anemia and 154 with hypertension, conditions that were subsequently managed through timely referrals and treatment. The program also achieved near-universal post-HIV test counseling and high follow-up rates, while community-based health education improved knowledge of safe birthing practices. In the US, the efforts of Sister Bernadette Kenny, a nurse practitioner who launched the Health Wagon program in a rural county in southwest Virginia in 1980 were reported as helping to lower the infant mortality in the service area [37].

Vaccination initiatives with substantial coverage were also a frequently reported metric across the literature as evidence of program success in improving health outcomes. For example, a study from the Netherlands reported that a mobile vaccination program serving rural communities during the COVID-19 pandemic was associated with a 14.5-fold increase in vaccination rates compared to baseline [38].

The Project Rural Recovery (PRR) MHC program in Tennessee provided data indicating improvements in chronic disease management outcomes. By collecting longitudinal clinical data across multiple patient encounters, PRR was able to document improvements in cardiometabolic health with clients demonstrating reductions in systolic (60%) and diastolic blood pressure (62%), and almost half of overweight or obese clients experiencing a reduction in BMI at their reassessment [28]. Similarly, a study of MHC programs in China reported enhanced continuity of care that facilitated more sustained engagement and management of chronic conditions, such as diabetes, hypertension, and hypothyroidism, among rural residents [39]. Between 2021 and 2023, the programs’ intelligent mobile clinic model documented 267,609 chronic disease follow-up encounters as well as delivery of 201,144 prescription services. While these metrics suggest improved chronic disease management and serve as a proxy indicator for medication continuity and adherence, we noted they were essentially program utilization metrics and patient outcome experiences rather than standardized quantitative clinical outcomes (e.g., changes in HbA1c or blood pressure values). This finding illustrates a common limitation in patient outcome measures across the documents reviewed. Overall, most of the rural MHC programs primarily reported the number of preventive visits and health screenings as evidence of improved clinical and health outcomes, yet the reporting of actual data showing measurable change in clinical outcomes was quite limited.

#### 4.2.2. Theme 2: High Patient Satisfaction and Perceived Quality of Care

While the relationship between patient satisfaction and perceptions of care quality to patient outcomes are complex, we decided to include them in this category because there is evidence that patients who are unsatisfied or believe services are poor quality may opt to not use them. Conversely, patient satisfaction with services can contribute to higher return visit rates as well as general community acceptability. Therefore, both positive or negative perceptions of care may indirectly influence service utilization and subsequently individual clinical and population health outcomes [40].

Across the MHC programs and settings in our review, patient satisfaction was consistently reported as high. Within this general finding, three sub-themes emerged: (1) appreciation for accessible, community-based care; (2) high perceived quality of interactions with staff and providers; and (3) strong trust and repeat utilization. While accessibility was discussed in the previous section, in that context it was from the perspective of program administrators. Here, the focus is on patients’ reported experiences of accessibility in relation to their satisfaction with the MHC program.

In the literature reviewed, patients consistently valued the accessibility and convenience of MHCs. For example, in Plan A Health, 95% of patients said the clinic made it easier to receive care, and nearly half would not otherwise have accessed services [41]. More than 90% of patients in Ohio University’s programs reported similar experiences, and cancer screening programs in rural Canada reported over 90% of patients felt the MHC increased their access to care [27,42]. Collectively, these data indicate that increasing access to care is an important factor in patient satisfaction with rural MHCs.

The literature also suggests that strong patient–provider relationships and perceived quality of care contribute to rural patient satisfaction with MHC services overall. Across the literature, patients frequently described MHC staff with terms such as respectful, compassionate, and attentive. Clemson Rural Health patients noted that providers “take the time to listen” and “really care,” while 97% of University of Iowa Mobile Clinic patients rated their care as “excellent” or “good,” with almost 90% reporting that all of their questions were addressed [30,43].

Lastly, the literature also linked high patient satisfaction with services to repeat engagement. For example, Iowa’s University Mobile Clinics reported over half of visits were from returning patients, with 6% seeking monthly care [30]. Similarly, in Nigeria’s Katsina State, over half of the recorded visits were from returnees, indicating ongoing patient engagement [44]. Traverse City’s MHC also reported almost 1000 visits from 400 individuals over 2 years—despite the unstable living conditions of the population served—as a sign of sustained engagement [22]. Finally, Project Rural Recovery relayed that 35% of their Year 4 clients returned for multiple visits, in spite of transportation and communication barriers [28]. These data suggest that when patients are satisfied with mobile services, they are more likely to return for ongoing care, which presumably contributes to better long-term patient outcomes.

#### 4.2.3. Theme 3: Broader Individual and Community-Level Benefits

Beyond clinical outcomes and patient satisfaction, many of the mobile health programs represented in our review reported broader individual and community impacts, including in education and general well-being. The literature described educational benefits ranging from enhancement of health professions student experience to local school and youth engagement, as well as promotion of health literacy across the community at large.

The Clemson University Impact Report mentions health professions students gaining clinical skills, cultural competence, communication, and confidence in working with underserved communities through participation in their mobile program [34]. The students themselves described their deeper understanding of social determinants of health and greater preparedness for future healthcare careers, suggesting MHCs can serve as a valuable training environment. Through their LION clinic, Penn State University also has created a mobile health model that couples hands-on medical education with community service to increase access to care [45]. School-based and youth-targeted mobile programs, such as Plan A Health and Tsehootsoo Medical Center’s teen wellness clinics, also reported participants were exposed to healthcare careers, supporting long-term pipeline development [41,46].

The literature also indicates that MHCs increase health literacy at the community level. Many MHC programs reported providing health education, including in areas such as nutrition, chronic disease self-management, reproductive health, and preventive care, which improves patients’ understanding of their health and adherence to recommendations. In addition to stabilizing chronic conditions, patients reported mobile programs supported their overall well-being. For example, patients expressed that if Plan A had not come to their town, they would not have gone for care elsewhere, suggesting not only improved access but also reduced health-related anxiety [41]. Similarly, CUAMM clinics in Southern Italy reported reduced stress related to health management and stronger motivation for maintaining healthy behaviors among migrant patients served by the program [47].

Overall, our literature review indicates rural MHCs can deliver benefits that extend beyond clinical outcomes, including by promoting education and health workforce development, reducing stress and increasing the ability to manage chronic conditions, all of which can contribute to better quality of life for individuals and overall community health.

### 4.3. Return on Investment (ROI) and Program Sustainability

Twenty-one of the 34 documents reviewed included data or information on ROI or sustainability. This included a mix of qualitative and quantitative data. We grouped the findings into two overarching categories: (i) ROI focusing on direct and indirect economic impacts of rural MHCs; and (ii) sustainability, which included mobile clinic operations, community acceptance, and long-term integration of the mobile clinic program into rural communities. Overall, the available information in this category was limited and varied widely across the documents, making it difficult to draw strong, evidence-based conclusions about the ROI and sustainability of rurally deployed MHC programs. However, we were able to identify four themes within this category in the literature reviewed.

#### 4.3.1. ROI

##### Theme 1: Cost-Effectiveness Compared with Traditional Healthcare Delivery

As mentioned, while many studies have shown MHCs can be a cost-effective approach to healthcare delivery compared with traditional brick-and-mortar facilities broadly, our literature review of programs that specifically serve rural populations both in the US and internationally appears to align with this general finding. More than half of the documents in our review that included some type of ROI data reported mobile services deliver comparable care with lower fixed costs than their non-mobile counterparts. As one example, Attipoe-Dorcoo found the overall cost of care provided via mobile clinics across several states was lower than per-patient Medicare cost benchmarks at Federally Qualified Health Centers [31]. La Clínica, an MHC program that operates in rural Oregon, reported their mobile teams used provider time more efficiently and improved operational productivity compared to brick-and-mortar facilities [48]. Additionally, vehicle-based health services in rural Sub-Saharan Africa reported substantially less upfront capital investment, an important consideration for resource-constrained regions [24].

Quantitative data supporting MHCs as more cost-effective than traditional forms of healthcare delivery in rural areas were also identified, with some evidence of positive financial growth and viability. For example, the Clemson Rural Health Innovation project reported a 9.4% increase in patient revenue, an average of $12 saved for every $1 invested, and $1600 saved per patient [43]. Ohio University’s Community Health Programs also claimed a substantial ROI, generating approximately $32 in social and economic value for every $1 invested in rural mobile health services [27].

##### Theme 2: Avoidance of Emergency Department Visits

In rural populations, emergency department utilization has increased disproportionately over the past decade, particularly among adults aged 18–64, Medicaid beneficiaries, and uninsured patients. In rural settings, many ED visits are low-acuity or better suited for ambulatory care, reflecting gaps in timely outpatient and primary care access [49]. A national study by the Mobile Health Map estimated that mobile clinics across all settings prevent over 11,000 ED visits annually [12]. In our review, avoidance of ED visits was a common metric mentioned by rural MHC programs to support the ROI. For example, the Clemson Rural Health Innovation program reported its mobile health clinics prevented an estimated 600 ED visits annually for each clinic [43]. Similarly, Project Rural Recovery (PRR) in Tennessee reported that across their mobile clinics, the average cost per visit was $600, compared with $1883 for an uninsured emergency department visit [28]. Additionally, 10–12% of PRR patients reported they would have visited the ED if mobile clinic services had not been available. These findings suggest MHCs contribute economic value as well as alleviate strain on safety-net EDs by improving access to primary and preventive care for hard-to-reach populations.

##### Theme 3: High Fixed Costs Offset by Multifunctionality

Across many rural MHC programs, both in the U.S. and abroad, mobile clinics were characterized by high fixed operating costs, which included expenses such as staff salaries, vehicle maintenance, and fuel for long-distance travel. For example, in rural South Africa, an MHC program that provided reproductive and primary health care for women and their partners reported fixed costs made up 94% and 85% of annual spending in two rural districts, respectively, primarily from staff salaries [18]. However, the literature also revealed that costs associated with adding or expanding services were marginal and helped offset high fixed costs. For example, in the previous example, adding primary care and sexual and reproductive health services required minimal additional clinical supplies but made a broader range of services available without significantly raising operational costs. Based on the cost analysis of specific services delivered, the author concluded that “where patient volumes do not exceed the capacity of the nurse offering services, incorporating multiple SRH [sexual and reproductive health] and other primary care services within a program for cervical cancer screening is one way to potentially expand access to a broader range of services without added costs” [18] (p. 10). In addition, increasing the multifunctionality of care delivery methods (such as integrating telemedicine or shared EMRs with distant referral centers) was also noted to increase the viability of MHCs in rural settings [21,43].

##### Theme 4: MHC Costs Increase with Lower Patient Density

When evaluating the cost-effectiveness of MHCs in rural regions, population density and distance traveled were noted to be important factors. In the Central Karoo region of South Africa, per-patient visit costs were higher in areas with lower population density [18]. Although this effect was quantified in only one study, it illustrates how geographic distribution, long travel distances between towns, and seasonal fluctuations in patient visits can influence ROI for rural mobile clinics.

#### 4.3.2. Sustainability

##### Theme 1: Patient and Community Acceptance

The literature reviewed suggests community uptake of rural MHC programs can enhance cost-effectiveness. In one article that described an MHC program implementing prenatal care and STI prevention in Mysore, India, community acceptance and consistent patient turnout were reported as indicators of long-term program viability [36]. Additionally, the authors noted that by integrating mobile health into rural and underserved regions, this program generated a scalable model for prenatal and infection prevention care.

Mobile Health Central in rural Michigan extrapolated from data on their last 10 years of operation to project a decrease in future healthcare expenditures and improved population health that relied on continued engagement of local communities with the mobile program [50]. Both the RAMS Know Healthcare on Wheels program in North Carolina and La Clínica in Oregon echoed the notion that their MHC programs promote long-term community health for underserved populations, which may help reduce overall healthcare costs in those areas [29,48].

Finally, a study on vaccination rates in rural communities in the Netherlands noted a key to the success and cost-effectiveness of the MHC program was its sustained long-term program implementation [38]. On a larger scale, Ohio University’s Community Health Programs, which include both MHCs and brick-and-mortar facilities, reported their long-standing operations in Southeast Ohio generated approximately $63 million of social and economic value annually [27]. These documents support the assertion that consistent community engagement and utilization contribute to the financial sustainability of rural MHCs.

##### Theme 2: Funding for Sustaining Rural MHC Operations

Many MHCs serving rural populations were found to depend on strategic partnerships and diverse funding sources to sustain their programs. Support from state agencies, hospital systems, and federal grants targeting rural health disparities, as well as donations of money, medications, supplies, and even vehicles, were common across the programs reviewed. For example, the Health Wagon in Appalachia initially received $20,000 in donations to support its operations, while Mobile Medical Units in Michigan secured a $1 million HRSA grant alongside additional contributions from local healthcare systems [50,51]. Similarly, Plan A mobile clinics operating in the Mississippi Delta and Southwest Georgia reported receiving $2 million in donations in 2023 and an additional $200,000 in grants in 2024 [41]. Programs with strong institutional partnerships, such as with the private sector, local universities, health systems, and/or community organizations, reported better long-term viability, including by capitalizing on shared staff, training resources, and operational support through these partnerships. Some programs, like the RAMS Know Healthcare on Wheels in North Carolina, also mobilized volunteer networks and local partnerships to expand their reach and maintain services while reducing operational costs [29]. Overall, the available evidence highlights that diverse funding and collaboration across sectors are vital to sustaining rural mobile health programs.

##### Theme 3: Building Local Rural Health Infrastructure

Several documents emphasized the value of MHCs for strengthening local infrastructure and building the capacity of rural healthcare systems. For example, an MHC program in rural Mysore, India, trained community health workers and retained health professionals, which in turn supported consistent preventive care efforts and yielded a scalable model for bringing prenatal and infection prevention services to other underserved regions [36]. In addition, several mobile health programs in the U.S. were integrated into academic institutions, which was noted to help with continuity of care, a known challenge for many rural populations [29,43,50].

## 5. Discussion

Our review of the published and grey literature on mobile health programs that serve rural populations reveals that MHCs are a promising strategy for addressing healthcare access barriers and improving health outcomes in underserved rural communities. However, the strength of the evidence supporting these conclusions varied considerably across the 34 documents we reviewed, reflecting both the diversity of programs and the heterogeneity of their data collection and reporting practices.

### 5.1. Evidence for Increased Access

The literature consistently demonstrated that MHCs successfully reach rural populations facing geographic, transportation, and economic barriers to care. Programs reported serving patients who traveled long distances, lacked reliable transportation, or could not afford traditional healthcare services. The widespread practice of providing free or low-cost services combined with bringing services to patients in remote locations clearly expanded access for vulnerable populations.

However, a key finding of our review is that much of the available access data consisted primarily of patient visit counts and service utilization metrics. While these numbers provide valuable information about program reach and activity, they were often conflated with actual increases in healthcare access without additional supporting evidence. This distinction is particularly important given that approximately half of the documents reviewed came from grey literature—annual reports, webpages, and program materials primarily designed to communicate success to funders and the public rather than to advance scientific knowledge. In this context, patient visit data serves as a simple, easily captured metric that demonstrates program activity and justifies continued funding, but may not fully capture whether services truly filled previously unmet healthcare needs or simply shifted care delivery from one setting to another. 

The literature also revealed that multi-service integration has become standard practice among rural MHCs rather than an innovative approach. Programs consistently bundled primary care, chronic disease management, preventive services, reproductive health, and behavioral health rather than offering single-purpose services as is common in many urban MHC programs. This finding makes practical sense: unlike high-density urban areas where populations can support specialized mobile services, rural communities often lack access to any healthcare, making comprehensive service delivery both clinically appropriate and economically necessary for program sustainability. For this reason, the finding that a “one-stop-shop” approach was common among rural MHC programs was not surprising.

### 5.2. Patient Outcomes: Promise and Evidence Gaps

The literature consistently reported delivery of preventive services such as vaccinations, health screenings, and chronic disease management visits as evidence of improved outcomes. Several programs documented impressive reach; however, our review identified a significant gap between reported service delivery and documented clinical outcome improvements. Most programs equated the provision of services with improved outcomes without presenting data showing measurable clinical changes. Notable exceptions included programs that tracked longitudinal clinical metrics such as blood pressure reductions, BMI changes, or specific disease management indicators over multiple patient encounters. The scarcity of such data represents a critical gap in evidence demonstrating that rural MHCs are effective in improving health outcomes.

Patient satisfaction data was consistently high across programs and settings, with patients valuing rural MHCs’ accessibility, convenience, and quality of interactions with providers. Multiple programs reported high repeat visit rates, suggesting sustained engagement and trust. While patient satisfaction does not directly measure clinical outcomes, it does serve as an important indicator of program acceptability and may influence health-seeking behaviors and treatment adherence, potentially contributing to improved long-term health outcomes. The literature also revealed broader community-level benefits including enhanced health professions education, health literacy, and community capacity to address health challenges. These effects, while difficult to quantify, suggest that rural MHCs may contribute to strengthening rural health infrastructure in ways that extend beyond direct patient care.

### 5.3. Return on Investment and Sustainability: Promising but Incomplete Evidence

Our review revealed that data on ROI and financial sustainability for rural MHCs was highly variable in quality and comprehensiveness. Across programs, vehicle-based services were found to require lower upfront capital investment than brick-and-mortar facilities; however, high fixed operational costs present a financial challenge for rural MHCs. Multi-service integration was a common strategy to offset these expenses, as the cost of adding services was typically low and was also appropriate for meeting the needs of rural populations. This finding has important implications for program design: rural MHCs appear to be most viable when offering comprehensive services rather than single-purpose care.

Avoidance of emergency department visits emerged as a common metric for demonstrating economic value. Given the high cost of ED care and the documented increase in low-acuity ED utilization in rural areas, this trend represents both economic benefit and relief for strained safety-net hospitals. However, some of these estimates appeared to rely on patient self-report of what they would have done without MHC access rather than rigorous comparison data.

An important finding regarding cost-effectiveness was that per-visit costs increase in areas with lower population density due to longer travel distances and potentially lower patient volumes per site. This has significant implications for the viability of MHCs in frontier or extremely rural areas and suggests that program success may be highly dependent on careful selection of service locations and populations.

Finally, the literature consistently revealed that diverse, sustained funding from multiple sources—including federal and state grants, hospital system partnerships, private donations, and institutional support—is essential for long-term program sustainability. Programs with strong institutional partnerships, particularly with academic institutions and healthcare systems, reported better long-term viability through shared resources, training support, and operational infrastructure.

## 6. Limitations

A fundamental limitation of this review stems from the nature of the literature itself. With approximately half of the documents coming from grey literature designed primarily for advocacy and fundraising rather than scientific inquiry, much of the available evidence lacks the rigor necessary to definitively establish MHCs’ effectiveness in improving rural health outcomes or demonstrating return on investment. While this literature provides valuable insight into program operations and perceived impact, the frequent conflation of service delivery metrics with actual access and health outcome improvements represents a significant evidence gap.

Additionally, our search strategy presents some limitations. We recognize that our Boolean search string relied heavily on MeSH terminology, which may have excluded recently published that were not yet indexed with MeSH terms. Additionally we did not use wildcards to capture plural forms and term variations. However, given our knowledge of the extant literature on MHCs and the dearth of published literature on rural health overall, we do not believe these limitations caused a sizeable amount of literature to be overlooked.

## 7. Implications and Recommendations

Despite these limitations, the available evidence suggests that mobile health clinics represent a promising strategy for expanding primary and preventive healthcare access in rural populations. The convergence of findings across diverse domestic and international settings, consistent reports of high patient satisfaction and repeat utilization, and success in reaching underserved populations all lean toward support for continued investment in and expansion of rural MHC programs.

Several key recommendations emerge from this review that should guide future rural MHC program development. First, multi-service integration appears essential for both meeting rural population health needs and achieving financial sustainability. Incorporating technological innovations such as telehealth capabilities and electronic health record integration may enhance program impact by expanding access to specialty care, supporting rural MHC providers, and improving care coordination. Third, diverse funding partnerships are critical for long-term viability. Finally, strong community engagement and acceptance are central to program success and sustainability.

Future research on MHCs should prioritize longitudinal studies with robust comparison groups that can establish causal relationships between MHC services and health outcomes. Studies should move beyond reporting visit counts and service utilization to measuring specific clinical outcomes, conducting rigorous cost-effectiveness analyses, and examining long-term impacts on population health and healthcare utilization patterns. Research is also needed to identify which service delivery models, operational strategies, and funding approaches best support sustainable rural MHC programs in diverse geographic and demographic contexts. Patient and provider acceptability and satisfaction with service delivery that compares MHCs with traditional brick-and-mortar settings is another area that warrants study, as this aspect of clinical care is often overlooked in the face of limited access. Partnership between rural MHC programs and academic institutions with capacity for conducting such rigorous research could be a practical strategy for advancing these research goals.

## 8. Conclusions

Mobile health clinics serving rural populations demonstrate promise as a strategy for addressing healthcare access barriers and improving health outcomes in underserved communities. The available evidence, while limited by methodological heterogeneity and reliance on grey literature, consistently shows that MHCs successfully reach rural populations, deliver valued services, and may provide cost-effective care compared to traditional delivery models. However, significant evidence gaps remain, particularly with rigorous demonstration of improved clinical outcomes and return on investment. Addressing these gaps through well-designed research studies should be a priority as the field moves forward. With continued innovation, diverse funding support, and commitment to evaluation, mobile health clinics have the potential to play an increasingly important role in strengthening rural healthcare infrastructure and reducing persistent rural–urban health disparities.

## Figures and Tables

**Table 1:** Documents by region.

Region	Number of Studies	Percentage
North America	16	47.1%
Africa	6	17.6%
Europe	3	8.8%
Asia	3	8.8%
South America	1	2.9%
Middle East	1	2.9%
Multiple countries (in Africa)	1	2.9%
TOTAL	34	100%

**Figure A1:**
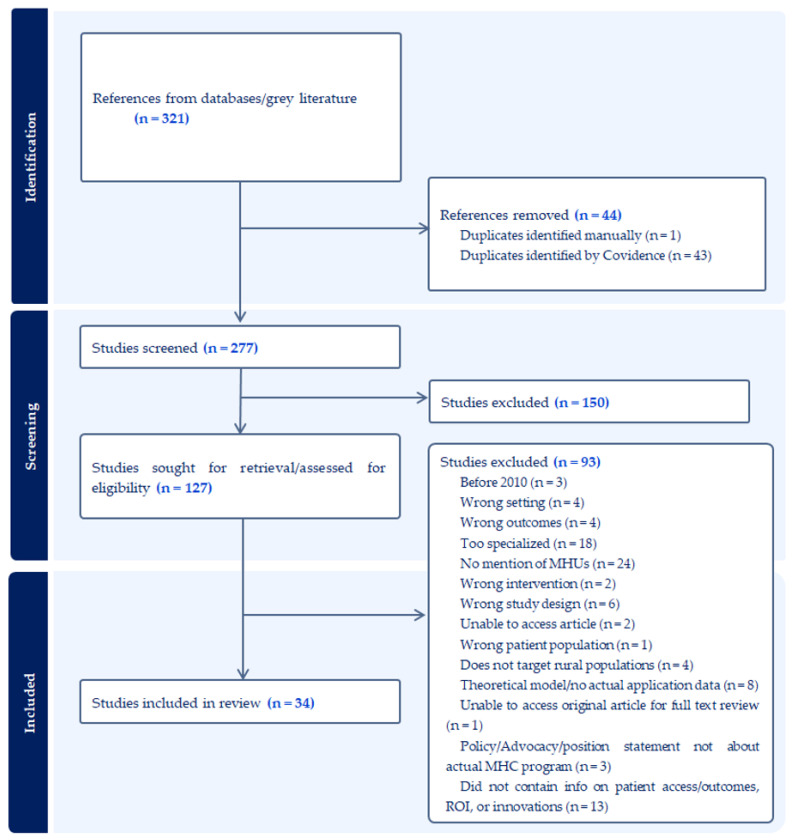
PRISMA flowchart for scoping review and study selection.

## Data Availability

No new data were created or analyzed in this study. Data sharing is not applicable to this article.

## Supplementary Materials

The following supporting information can be downloaded at: https://www.mdpi.com/article/10.3390/ijerph23050558/s1, Table S1: Preferred Reporting Items for Systematic reviews and Meta-Analyses extension for Scoping Reviews (PRISMA-ScR) Checklist [52]; Table S2: Characteristics of Sources of Evidence.
