# Supplementary material for: What Do We Know About Rural Mobile Health Clinics? A Scoping Review
Source: Int J Environ Res Public Health. 2026 Apr 25;23(5):558. doi: 10.3390/ijerph23050558 (PMC13207173; doi:10.3390/ijerph23050558)
Supplement: Supplementary file 1 [file ijerph-23-00558-s001.zip › ijerph-4099110 Table S1.pdf]

Preferred Reporting Items for Systematic reviews and Meta-Analyses extension for Scoping Reviews (PRISMA-ScR) Checklist

SECTION	ITEM	PRISMA-ScR CHECKLIST ITEM	REPORTED ON PAGE #
**TITLE**			
Title	1	Identify the report as a scoping review.	What Do We Know About Rural Mobile Health Clinics? A Scoping Review
**ABSTRACT**			
Structured summary	2	Provide a structured summary that includes (as applicable): background, objectives, eligibility criteria, sources of evidence, charting methods, results, and conclusions that relate to the review questions and objectives.	Rural communities face significant healthcare access barriers that contribute to persistent health disparities. Mobile health clinics (MHCs) have emerged as a promising strategy for expanding healthcare access, yet their effectiveness in rural settings remains understudied. This aim of this literature review was to determine whether mobile health clinics: (a) increase access to care; (b) improve patient outcomes; and (c) can support the return on investment (ROI) and be financially and programmatically sustainable in rural populations.<br>We conducted a comprehensive search of peer-reviewed literature and grey literature sources. Systematic screening yielded 34 documents for full analysis. Thematic analysis was conducted across three domains: patient access, patient outcomes, and ROI/sustainability. All 34 documents provided data on patient access, with common themes including expanded service utilization, multi-service integration, overcoming geographic and transportation barriers, and improved healthcare affordability. Thirty-two documents addressed patient outcomes, reporting improvements in preventive care delivery, chronic disease management, and high patient satisfaction. Twenty-eight documents included ROI/sustainability information, with evidence suggesting cost-effectiveness particularly through emergency department visit avoidance and multi-service integration. Across the literature reviewed, the quality of evidence varied considerably, yet we concluded mobile health clinics

St. Michael's
Inspired Care.
Inspiring Science.

SECTION	ITEM	PRISMA-ScR CHECKLIST ITEM	REPORTED ON PAGE #
			demonstrate promise for expanding healthcare access and improving outcomes in rural populations. Key success factors include multi-service integration, diverse funding partnerships, technological integration, and strong community engagement. More rigorous research with longitudinal clinical outcome measures and robust economic analyses is needed.
**INTRODUCTION**			
Rationale	3	Describe the rationale for the review in the context of what is already known. Explain why the review questions/objectives lend themselves to a scoping review approach.	Mobile health clinics (MHCs) have been garnering growing attention in the United States and globally as a promising healthcare delivery strategy for bridging gaps in access, especially among rural and other hard-to-reach populations. Although MHCs have been shown to operate successfully in a variety of settings, including many urban areas, their impact in rural communities is less well understood. Key questions about whether MHCs can expand access to care, improve patient outcomes, and provide a cost-effective approach to healthcare delivery in rural areas are important for determining whether the investment of time, money and other resources into such programs is warranted. The aim of this literature review is to examine current evidence on the effectiveness of MHCs in bridging gaps in primary care access and in improving health outcomes in rural populations, as well as on the return on investment (ROI) and financial sustainability. Our familiarity with the field of mobile healthcare and with the existing published literature on mobile health clinics broadly led us to decide a scoping review was most appropriate for assessing the evidence on their use in rural areas.
Objectives	4	Provide an explicit statement of the questions and objectives being addressed with reference to their key elements (e.g., population or participants, concepts, and context) or other relevant key elements used to conceptualize the review questions and/or objectives.	RQ: Are mobile health clinics an effective way to address the primary health care needs of rural populations in the United States? Specifically, we sought to identify existing evidence on whether mobile health clinics: a) increase access to care; b) improve patient outcomes; and c) can support the return on investment (ROI) and

SECTION	ITEM	PRISMA-ScR CHECKLIST ITEM	REPORTED ON PAGE #
			be financially sustainable in rural populations.
**METHODS**			
Protocol and registration	5	Indicate whether a review protocol exists; state if and where it can be accessed (e.g., a Web address); and if available, provide registration information, including the registration number.	A review protocol does not exist
Eligibility criteria	6	Specify characteristics of the sources of evidence used as eligibility criteria (e.g., years considered, language, and publication status), and provide a rationale.	Inclusion criteria: Limited to articles published in English since 2009. Both U.S.-based and international mobile health programs were included. Additionally required the mobile health clinic program to serve a rural population and deliver general healthcare services, such as primary care, chronic disease management, health screenings, or vaccinations. Exclusion criteria: Programs that only offered specialty services (e.g., only mammography, HIV-care, dental services, or mental health/substance use disorder services). Programs that integrated such services into a broader primary care model were retained for full text review.
Information sources*	7	Describe all information sources in the search (e.g., databases with dates of coverage and contact with authors to identify additional sources), as well as the date the most recent search was executed.	Formal search: PubMed, Social Science Premium Collection, Business Source Complete, and ProQuest Healthcare Administration. The most recent search of databases was conducted in July 2025.<br>For grey literature: Began with list of self-reported mobile health clinics obtained from the Mobile Health Map database and selected clinics that self-identified as serving exclusively rural, frontier, or rural/frontier communities, and excluded those operating in suburban or urban settings. Programs that provided only specialized services (e.g., mammography, dental-only services) were removed from the list. The remaining programs were screened for eligibility through a two-step process. First, we carried out a preliminary verification of clinic type and activity using an AI-based tool (ChatGPT). Second, a reviewer manually confirmed each clinic's status, with uncertain cases reviewed by a second reviewer (KS) or

St.Michael's
Inspired Care.
Inspiring Science.

SECTION	ITEM	PRISMA-ScR CHECKLIST ITEM	REPORTED ON PAGE #
			discussed with the research team during a meeting
Search	8	Present the full electronic search strategy for at least 1 database, including any limits used, such that it could be repeated.	In PubMed, we employed the following MeSH-based terms to generate an initial body of articles:("Rural Health"[MeSH Terms] OR "Rural Population"[MeSH Terms]) AND ("Mobile Health Units"[MeSH Terms] OR "mobile health unit" OR "mobile health clinic") AND "Health Services Accessibility"[MeSH Terms] AND "Delivery of Health Care"[MeSH Terms]. We also used variations such as: ("Rural Health"[MeSH Terms] OR "Rural Population"[MeSH Terms]) AND ("Mobile Health Units"[MeSH Terms] OR "mobile health unit" OR "mobile health clinic"). To broaden our search and capture studies with diverse methodological frameworks, we also searched using keyword combinations such as: ("Rural Health"[MeSH Terms] OR "Rural Population"[MeSH Terms]) AND ("Mobile Health Units"[MeSH Terms] OR "mobile health unit" OR "mobile health clinic") NOT (mammogram OR mammography OR breast OR asthma OR behavioral health OR psychiatric OR psychology OR mental health OR mental illness OR dental OR dentist OR oral OR (maternal AND infant) OR (maternal AND child) OR pediatrics OR child OR infant OR natal OR neonatal OR sexual OR reproductive OR vision OR eye).
Selection of sources of evidence†	9	State the process for selecting sources of evidence (i.e., screening and eligibility) included in the scoping review.	Documents retained for full-text review were screened to confirm they included data on at least one of our research foci: impact on healthcare access and/or patient outcomes, or information about ROI or financial sustainability. Additionally, all documents that met inclusion criteria were reviewed to verify that the mobile health program was delivered entirely or in some part out of some type of vehicle (in contrast to programs operating out of a non-permanent, non-clinical space, such as a community center, church, school, or that were solely digitally based).
Data charting process‡	10	Describe the methods of charting data from the included sources of evidence (e.g., calibrated forms or forms that have been tested by the team	Following the search of the databases and grey literature, documents that met inclusion criteria were uploaded to Covidence. Titles and abstracts were then independently reviewed by two team

SECTION	ITEM	PRISMA-ScR CHECKLIST ITEM	REPORTED ON PAGE #
		before their use, and whether data charting was done independently or in duplicate) and any processes for obtaining and confirming data from investigators.	members to assess relevance against our predefined inclusion and exclusion criteria. Each document retained for full-text review was then reviewed by one team member who placed pertinent information from each document into an evidence table spreadsheet with categories of patient outcomes, patient access, economic sustainability/ROI. No additional data were obtained or confirmed by document authors.
Data items	11	List and define all variables for which data were sought and any assumptions and simplifications made.	Access to healthcare services<br>Patient outcomes<br>Return on investment/sustainability
Critical appraisal of individual sources of evidence§	12	If done, provide a rationale for conducting a critical appraisal of included sources of evidence; describe the methods used and how this information was used in any data synthesis (if appropriate).	n/a
Synthesis of results	13	Describe the methods of handling and summarizing the data that were charted.	Data charted into the evidence table spreadsheet were discussed by the research team and described in the Results section of a manuscript developed for submission. Further communication among team members about these findings occurred in conjunction with the writing and final review of the manuscript prior to submission to the journal
**RESULTS**			
Selection of sources of evidence	14	Give numbers of sources of evidence screened, assessed for eligibility, and included in the review, with reasons for exclusions at each stage, ideally using a flow diagram.	321peer-reviewed and grey literature articles were uploaded into Covidence for systematic screening. After removing 44 duplicates, 277 articles/artifacts remained. Titles and abstracts were independently reviewed by two team members to assess relevance against our predefined inclusion and exclusion criteria. Articles that were irrelevant or did not meet our criteria, such as those not focusing on MHUs, not serving rural populations, or outside the date range, were excluded, resulting in a total of 127 articles for full-text review. During full-text review, studies were further screened for relevance. Articles were excluded if they did not mention MHUs, focused exclusively on specialty services, or had no data on patient access, outcomes, or ROI/financial sustainability. Following this step, 34 studies remained

SECTION	ITEM	PRISMA-ScR CHECKLIST ITEM	REPORTED ON PAGE #
			for detailed data extraction. A PRISMA diagram illustrates this process.
Characteristics of sources of evidence	15	For each source of evidence, present characteristics for which data were charted and provide the citations.	Included as supplementary table to the manuscript
Critical appraisal within sources of evidence	16	If done, present data on critical appraisal of included sources of evidence (see item 12).	n/a
Results of individual sources of evidence	17	For each included source of evidence, present the relevant data that were charted that relate to the review questions and objectives.	See manuscript
Synthesis of results	18	Summarize and/or present the charting results as they relate to the review questions and objectives.	All 34 documents included some type of data/information about access to healthcare; 32 included information about patient outcomes; 28 included information about return on investment/sustainability of rural mobile health programs.
**DISCUSSION**			
Summary of evidence	19	Summarize the main results (including an overview of concepts, themes, and types of evidence available), link to the review questions and objectives, and consider the relevance to key groups.	In the category of Healthcare access, themes included: Patient Visits and Service Utilization; Multi-Service Integration; Overcoming Geographic, Transportation and Infrastructure Barriers; and Healthcare Affordability.<br><br>In the category of Patient Outcomes, themes included: Clinical and Preventive Health Outcome Improvements; High Patient Satisfaction and Perceived Quality of Care; and Broader Individual and Community-Level Benefits.<br><br>In the category of ROI/sustainability, themes included: Cost-Effectiveness Compared with Traditional Healthcare Delivery; Avoidance of Emergency Department Visits; High Fixed Costs Offset by Multifunctionality; Costs Increase with Lower Patient Density; Patient and Community Acceptance; Funding for Sustaining Rural MHU Operations; and Building Local Rural Health Infrastructure<br><br>Data from rigorous peer-reviewed research studies was limited but rather was largely

SECTION	ITEM	PRISMA-ScR CHECKLIST ITEM	REPORTED ON PAGE #
			from literature designed for other purposes (not research), such as program evaluations, webpages, news reports, etc. designed to communicate program successes to funders and the public.
Limitations	20	Discuss the limitations of the scoping review process.	A fundamental limitation of this review stems from the nature of the literature itself. With approximately half of the documents coming from grey literature designed primarily for advocacy and fundraising rather than scientific inquiry, much of the available evidence lacks the rigor necessary to definitively establish MHCs' effectiveness in improving rural health outcomes or demonstrating return on investment. While this literature provides valuable insight into program operations and perceived impact, the frequent conflation of service delivery metrics with actual access and health outcome improvements represents a significant evidence gap. Additionally, our search strategy presents some limitations. We recognize that our Boolean search string relied heavily on MeSH terminology, which may have excluded recently published that were not yet indexed with MeSH terms. Additionally we did not use wildcards to capture plural forms and term variations.
Conclusions	21	Provide a general interpretation of the results with respect to the review questions and objectives, as well as potential implications and/or next steps.	Mobile health clinics serving rural populations demonstrate considerable promise as a strategy for addressing healthcare access barriers and improving health outcomes in underserved communities. The available evidence, while limited by methodological heterogeneity and reliance on grey literature, consistently shows that MHCs successfully reach rural populations,

SECTION	ITEM	PRISMA-ScR CHECKLIST ITEM	REPORTED ON PAGE #
			deliver valued services, and may provide cost-effective care compared to traditional delivery models. However, significant evidence gaps remain, particularly with rigorous demonstration of improved clinical outcomes and return on investment. Addressing these gaps through well-designed research studies should be a priority as the field moves forward. With continued innovation, diverse funding support, and commitment to evaluation, mobile health clinics have the potential to play an increasingly important role in strengthening rural healthcare infrastructure and reducing persistent rural-urban health disparities.
**FUNDING**			
Funding	22	Describe sources of funding for the included sources of evidence, as well as sources of funding for the scoping review. Describe the role of the funders of the scoping review.	No external funding was obtained to support this Scoping Review.

JBI = Joanna Briggs Institute; PRISMA-ScR = Preferred Reporting Items for Systematic reviews and Meta-Analyses extension for Scoping Reviews.
* Where *sources of evidence* (see second footnote) are compiled from, such as bibliographic databases, social media platforms, and Web sites.
† A more inclusive/heterogeneous term used to account for the different types of evidence or data sources (e.g., quantitative and/or qualitative research, expert opinion, and policy documents) that may be eligible in a scoping review as opposed to only studies. This is not to be confused with *information sources* (see first footnote).
‡ The frameworks by Arksey and O'Malley (6) and Levac and colleagues (7) and the JBI guidance (4, 5) refer to the process of data extraction in a scoping review as data charting.
§ The process of systematically examining research evidence to assess its validity, results, and relevance before using it to inform a decision. This term is used for items 12 and 19 instead of "risk of bias" (which is more applicable to systematic reviews of interventions) to include and acknowledge the various sources of evidence that may be used in a scoping review (e.g., quantitative and/or qualitative research, expert opinion, and policy document).

From: Tricco AC, Lillie E, Zarin W, O'Brien KK, Colquhoun H, Levac D, et al. PRISMA Extension for Scoping Reviews (PRISMAScR): Checklist and Explanation. Ann Intern Med. 2018;169:467–473. doi: 10.7326/M18-0850.
