# Supplementary material for: What Do We Know About Rural Mobile Health Clinics? A Scoping Review
Source: Int J Environ Res Public Health. 2026 Apr 25;23(5):558. doi: 10.3390/ijerph23050558 (PMC13207173; doi:10.3390/ijerph23050558)
Supplement: Supplementary file 1 [file ijerph-23-00558-s001.zip › ijerph-4099110 Table S2.pdf]

Characteristics of Sources of Evidence.

Title	Authors	Country or region in which the study conducted	Study design or type of artifact (if not research study)	Patient access data	Patient outcomes data	Return on investment/sustainability	Aim of study
Cost Evaluation of Reproductive and Primary Health Care Mobile Service Delivery for Women in Two Rural Districts in South Africa	Schnippel, K., Lince-Deroche, N., van den Handel, T., Molefi, S., Bruce, S., Firnhaber, C	South Africa	Economic evaluation	☑	☐	☑	Evaluated the costs of van-based mobile reproductive and primary health care clinics in two rural South African districts. The clinics provided integrated sexual and reproductive health, HIV/TB services, and basic primary care.
An Overview of Costs, Utilization, Geographical Distribution & Influence of Mobile Clinics in Rural Healthcare Delivery in the United States	Attipoe-Dorco, S.	United States	Systematic review	☑	☐	☑	Described the utilization and costs of a sample of mobile clinics operating in Texas, North Carolina, Georgia, and Florida. The study also assessed the effects of mobile clinics in states with high numbers of uninsured patients and rural communities.
Driving Sustainable Development: The Power of Vehicle-Based Services in Rural Sub-Saharan Africa	Pizzinini, C.	Sub-Saharan Africa	Program evaluation	☑	☑	☑	Describes a vehicle-based service delivery program providing essential services to rural communities across sub-Saharan Africa. The program uses MHUs to transport people, goods, and service equipment to improve access to healthcare and other critical resources in areas with limited infrastructure.
Mobile outreach clinics for improving health care services accessibility in vulnerable populations of the Diffa Region in Niger: a descriptive study	Rabiou, L. M.	Niger	Descriptive comparative study	☑	☑	☑	Evaluates whether mobile outreach clinics could improve access to essential health services for vulnerable populations in Niger's Diffa Region
Implementing a Teaching Rural Mobile Health Clinic: Challenges and Adaptations	Brant, K., Segel, J. E., McShane, M. P., Rhubart, D., Kowalkowski, J., et al.	United States	Qualitative research	☑	☐	☐	
Developing an Intelligent Mobile Clinic—A Medical Vehicle for Improve Access to Healthcare in Remote Areas: Evidence From China	Chen, X., Huang, X., Xu, Y., Xu, J., Wang, Y., Ren, X., Zhu, X., Xie, X., & Yang, Y.	China	Program evaluation	☑	☑	☑	Use the application, effectiveness and difficulties of the intelligent mobile clinic model in remote mountainous areas to provide reference experience for other remote areas to improve the uneven medical resources and provide high-quality medical services.
Exploring Mobile Health Clinics: A Scoping Review	Sabo, K.	United States	Systematic review	☑	☑	☑	Thematic analysis of the state of MHCs in the US.
Mobile primary healthcare services and health outcomes of children in rural Namibia.	Aneni, E., De Beer, I., Hanson, L., Rijnen, B., Brenan, A. T., Feeley, F.	Namibia	Cohort study	☑	☑	☐	Assess the health changes of orphans, other vulnerable children, and non-vulnerable children attending a mobile primary health care clinic in rural Namibia, and to evaluate whether regular mobile clinic visits improved key health indices
From the trunk of a Volkswagen beetle: a mobile nursing clinic in Appalachia.	Snyder, A., Thatcher, E.	United States	Historical case report	☑	☑	☑	Documents and analyzes the history, development, and impact of the Health Wagon mobile clinic, focusing on how it increased access to healthcare in an underserved Appalachian region and its influence on health outcomes like infant mortality
Mobile clinic services to serve rural populations in Katsina State, Nigeria: perceptions of services and patterns of utilization.	Peters, G., Doctor, H., Afenyadu, G., Findley, S., Ager, A.	Nigeria	Cross sectional study	☑	☑	☑	Assess early evidence on how mobile clinics in Katsina State, Nigeria, improve access to primary health care in hard-to-reach rural communities. Data included key informant interviews with community leaders and providers, exit interviews with 455 service users, and a household survey of 564 women, alongside analysis of routine service records.
An Innovative Approach to Enhancing Access to Medical Screening for Miners using a Mobile Clinic with Telemedicine Capability.	Evans, K., Lerch, S., Wilson Boyce, T., Myers, O. B., Kocher, E., Cook, L. S., Sood, A.	Chile	Text and opinion	☑	☑	☑	
Implementing and sustaining a mobile medical clinic for prenatal care and sexually transmitted infection prevention in rural Mysore, India.	Kojima, N.	India	Case series	☑	☑	☑	
Barriers to access and organization of primary health care services for rural riverside populations in the Amazon.	Garnelo, L., Parente, R., Puchiarelli, M., Correia, P., Torres, M., & Herkrath, F.	Brazil	Qualitative research	☑	☑	☐	Identifies the strategies of access and utilization of primary health care services by assessing the strengths and limitations of the healthcare model offered by the FMU to reduce barriers to services and ensure the right to healthcare
Pilot Study to Assess the Feasibility of a Mobile Unit for Remote Cognitive Screening of Isolated Elderly in Rural Areas.	Zeghari, R.	France	Diagnostic test accuracy study	☑	☑	☑	Determine if there was a difference in neurological clinical assessment in a clinic or telemedical setting.
Mobile vaccination units to increase COVID-19 vaccination uptake in areas with lower coverage: a within-neighbourhood analysis using national registration data, the Netherlands, September-December 2021.	Lambooij, M. S.	Netherlands	Cohort study	☑	☑	☑	

Integrated access to cancer screening: expanding access for cervical and colorectal cancer screening in rural and remote Northern Alberta, Canada using a mobile service to bring cancer screening closer to home.	Wiseman, J., Patterson, K., Kliewer, G., Mueller, M., Mutti-Packer, S., Newsome, J., Lockerbie, S., Hauber, J., Schwann, M., Yang, H.	Canada	Non-randomised experimental study	☑	☑	☐	to evaluate the outcomes and impact the IACS initiative had on the communities and residents of Northern Alberta
Bridging Gaps in Migrant Healthcare: CUAMM's Experience from 13,103 Visits in Southern Italy.	De Virgilio Suglia C, Laforgia R, Schiavone M, Belfiore A, Laforgia N, Saracino A, Putoto G, Di Gennaro F.	Italy	Prevalence study	☑	☑	☐	This study investigates the health needs of migrant agricultural workers in Puglia, assesses the effectiveness of mobile clinics, and identifies systemic barriers to accessing care.
LION Mobile Clinic	McShane, M.	United States	Grey Literature: Webpage describing program	☑	☑	☐	
Mobile Health Central: 10 years of health on the road	Hodgkins, K., Zimmerman, G., Nelson, R.	United States	Text and opinion	☑	☑	☑	
A Mobile Clinic Set Out to Improve Reproductive Care for Women in the Mississippi Delta. The Problems It Found Went Much Deeper.	Gregory, V.	United States	News report	☑	☑	☐	
Augusta Health Receives 2024 CMS Health Equity Award		United States	News report	☑	☑	☐	A press release on the Augusta Health website announcing that Augusta Health received a 2024 CMS Health Equity Award from the Centers for Medicare & Medicaid Services.
Clemson Rural Health Innovation		United States	Website and program evaluation impact report	☑	☑	☑	
Mobile Health Program | Tséhootsooí Medical Center		United States	Website	☑	☐	☐	
Mobile Medical Unit		United States	Website	☑	☑	☑	
Ohio University's Community Health Programs has $63 million financial impact on Southeast Ohio		United States	Text and opinion	☑	☑	☑	
Project Rural Recovery - Delivering Mobile Integrated Care WhereTennesseans Live, Work, and Recover (Year 4 Annual Report)		United States	Annual Report	☑	☑	☑	
Our Impact - Health Wagon		United States	Webpage about overall program	☑	☑	☑	Webpage detailing information about overall program.
Plan A Health 2023 Annual Report	Weinberg, C.	United States	Annual Report	☑	☑	☑	Annual report summarizing progress, finances, and upcoming plans for the Plan A Health organization
Kuruman Community Trust Mobile Medical Clinic – Innovo Mobile Health Clinics		South Africa	Webpage describing program	☑	☑	☑	
Kefraya Mobile Medical Unit		Lebanon	Webpage description of program	☑	☐	☐	
Rural mobile clinic delivers care where care is hard to come by.		United States	Case report	☑	☑	☐	
Provision of Preventive Healthcare Services through a Mobile Health Clinic in East Winston-Salem, North Carolina.	Haynes, J. N.	United States	Text and opinion	☑	☑	☑	
Service Learning in Health Care for Underserved Communities: University of Iowa Mobile Clinic, 2019.	Palma, M. L., Arthofer, A., Halstead, K. M., Wahba, J. M., Martinez, D. A.	United States	Descriptive program evaluation	☑	☑	☐	
Mobile health clinics in a rural setting: a cost analysis and time motion study of La Clínica in Oregon, United States.	Higgins, A., Tilghman, M., Lin, T. K.	United States	Cost Analysis /Observational Program Evaluation	☑	☑	☑	
